# Large scale application of pulse oximeter and auscultation in screening of neonatal congenital heart disease

**DOI:** 10.1186/s12887-022-03540-7

**Published:** 2022-08-12

**Authors:** Yuqiang Huang, Shiqing Zhong, Xianmei Zhang, Linghui Kong, Wenli Wu, Shixia Yue, Ning Tian, Guanghua Zhu, Aiqin Hu, Juan Xu, Haijan Zhu, Airong Sun, Fangling Qin, Ziwen Wang, Shiqiang Wu

**Affiliations:** 1 Department of Pediatric Cardiothoracic Surgery, Linyi Maternal and Child Healthcare Hospital, Linyi City, Shandong Province 276016 People’s Republic of China; 2 Department of Ultrasound Diagnosis, Linyi Maternal and Child Healthcare Hospital, Linyi City, Shandong Province People’s Republic of China; 3 Department of Obstetrics, Linyi Maternal and Child Healthcare Hospital, Linyi City, Shandong Province People’s Republic of China; 4 Department of Neonatal Medicine, Linyi Maternal and Child Healthcare Hospital, Linyi City, Shandong Province People’s Republic of China

**Keywords:** Congenital heart disease, Pulse oximetry, Auscultation, Screening, Critical congenital heart disease

## Abstract

**Purpose:**

To conduct a retrospective evaluation of a large clinical implementation of combined pulse oximeter (POX) and cardiac auscultation as a fast-screening device for congenital heart disease (CHD).

**Methods:**

Every newborn in a large maternity healthcare center received auscultation and POX screening within 24 hours after delivery. When an abnormal heart murmur or SpO_2_ level was detected, an echocardiogram was ordered to confirm the diagnosis of CHD.

**Results:**

From January 1, 2018 to December 31, 2019, there were 44,147 livebirths at the studied hospital where 498 suspected CHD were identified: 27 newborns by POX screening and 471 by cardiac auscultation. The diagnosis was further confirmed in 458 neonates through echocardiogram. This result put forth an overall diagnosis rate of 92.0%. Cardiac auscultation detected the majority of CHD cases 438 (95.6%) while POX only screened 20 (4.4%) cases. Interestingly, no CHD case was detected by both auscultation examination and POX screening. Auscultation detected most of the common types of CHD, but POX excelled in identifying rare and critical cases. POX screening alone had a very low accuracy of 74.07% in positive predict value (PPV). On the other hand, auscultation functioned well in terms of PPV and negative predict value (NPV) (92.99 and 99.95%, respectively), but the addition of POX improved the overall screening performance resulting in 100% NPV. We also validate the finding with the data 6 months after the study period.

**Conclusion:**

Our study demonstrated that addition of pulse oximetry to routine cardiac auscultation could be used as an accurate and feasible screening for early screening of CHD in newborns in large-scale clinical practice.

## What is known

- ➓ Pulse-oximetry is safe, acceptable, non-invasive and effective.
- ➓ Pulse oximetry screening increases early diagnosis of major CHD as well as other important pathology with a very low false positive rate and minimal requirement for extra echocardiograms.
- ➓ Pulse-oximetry plus cardiac auscultation significantly improved the detection rate of major CHD in the early neonatal stage, with high sensitivity and a reasonable false-positive rate.

## What is new

- ➓ Screening with pulse oximetry or auscultation alone within the first 24 hours of life may not detect all cases of CHD.

## Introduction

Congenital heart disease (CHD) is the most common type of congenital malformations, with an overall prevalence of 6-10‰ in newborns, with ~ 2-3‰ severe cases [1]. About a quarter of CHD patients require surgery or catheterization in neonatal period, or early infancy [2]. Timely diagnosis and treatment can greatly improve the prognosis of patients [3]. Conversely, delayed diagnosis often leads to serious hypoxia, shock, acidosis, pneumonia, and other complications including death. Studies have reported that delayed diagnosis of critical congenital heart disease (CCHD) led to death in 1-2 of every 100,000 live births in the United States [4]. Prenatal obstetric ultrasound can only detect ~ 30% fetuses with CHD [5], but early neonatal diagnosis is still a big challenge.

Although echocardiography is the golden standard in CHD diagnosis, it usually takes more than 10 minutes to perform, and it is not practical to perform echocardiography for every newborn in areas where resource is limited. As an alternative, pulse oximetry (POX) is easy to operate and requires only 2 to 3 minutes to analyze the results. Besides, POX as an adjunct to current routine practice is likely to be a cost-effective strategy in the light of currently accepted thresholds [6]. It is highly specific in detecting CCHD with moderate sensitivity, and had been widely employed [7]. Since Dr. Guoying Huang introduced and established CHD screening system via POX in 2018, it has been gradually adopted in clinical practice in China [8].

As a large maternal and child healthcare facility, the studied hospital delivers more than 20,000 babies annually. Providing quick, convenient, and accurate screening tools for CHD detection among newborns are crucial for the local community in the absence of echocardiologist. Since 2018, this health center implemented a new strategy of combined auscultation and POX in CHD screening among newborns. When suspected CHD babies are identified by POX or auscultation, an echocardiography was requested for confirmation. This study was the first performance report from large-scale implementation of combined auscultation and POX in CHD screenings.

## Methods

### Study design and participants

We conducted this retrospective study, from January 1, 2018 to December 31, 2019, at Linyi Maternal and Child Healthcare Hospital. All consecutive newborns were eligible, irrespective of gestational age, NICU admission, symptom presence or prenatal diagnosis. This study was approved by institutional ethical review board of Linyi Maternal and Child Healthcare Hospital. Verbal, informed consent was obtained from the participants’ parents. The registry number is NCT05105880, 03/11/2021 (https://register.clinicaltrials.gov).

### Procedures

**Fig. 1:**
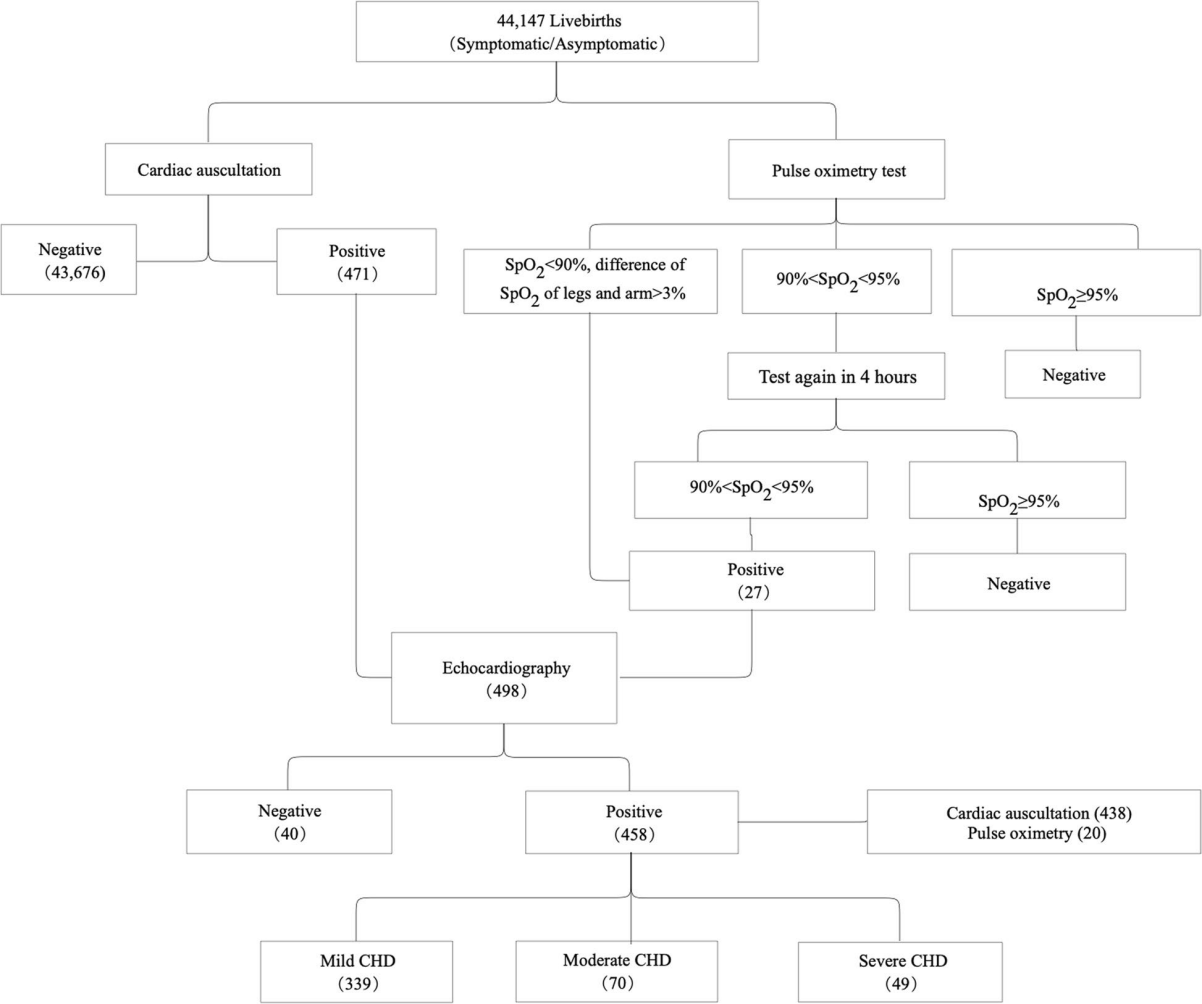
Screening process

All newborns would undergo POX and auscultation screening. Depending on the screening results, suspected-CHD neonates would then receive echocardiography confirmation (Fig. 1).

#### Cardio-auscultation

Pediatricians from Departments of Neonatal Medicine and Developmental Medicine are responsible for auscultation screening. A standard pediatric stethoscope (Model 3200, 3 M Littmann) was used. Cardiac auscultation was performed on the five points of auscultation (pulmonic, aorta, Erb’s point, tricuspid, and mitral) for each newborn under typical conditions of the general maternity ward, and the duration lasted 60 seconds per neonate. When abnormal murmur occurred, an echocardiography was requested to confirm if the newborn has CHD or not.

#### Pulse oximetry

Physicians from obstetrics department and nursing staff from neonatal department examined POX measurements using a pulse oximeter (Masimo, Irvine, CA, USA), with a multisite reusable or disposable sensor (Model 2329, Masimo). All babies were screened within 24 hours after birth. Pulse oximeter oxygen saturation (SpO_2_) was measured from the right hand and on either foot, and SpO_2_ levels were recorded on the nursing report sheet. If the measurement was less than 90% or the difference between two extremities was more than 3%, the baby was referred for echocardiography immediately. If one extremity’s measurement ranged between 90 and 94%, the clinician repeated the test on the baby 4 hours later. If measurements on both extremities were between 90 and 94%, the baby was referred for echocardiography.

#### Echocardiography

Physicians from Department of Ultrasonography confirmed the diagnosis of CHD among newborns screened by auscultation or POX through directing echocardiography. Pediatricians then referred the CHD results to cardiology center for an echocardiographic examination. After examination of cardiac position, atrioventricular morphology, valve morphology, and the connection between valves and large blood vessels, cardiac sonographer made the diagnosis of CHD according to the International Pediatric and Congenital Cardiac Code [9]. On the basis of the CHD severity classification recommended by Hoffman’s method, CHD cases were further classified into severe, moderate and mild groups, and prescribed treatment plans accordingly [10].

## Results

During 2-year study period, there were a total of 44,147 livebirths in the studied hospital. The demographic characteristics of the screened population were summarized in Table 1. The majority of the newborns were full term births delivered between 37 and 40 gestational weeks, with a median gestational age of 39 weeks. The median birth weight for newborn was 3420 g. All babies underwent screening within 24 hours after birth and cesarean section rate was 45.2%.

**Table 1:** Demographic characteristics of newborns

Variables	Total *N* = 44,147	Normal *N* = 43,689	Mild CHD *N* = 339	Moderate CHD *N* = 70	Severe CHD *N* = 49
Gestational age (weeks)					
< 37	2691 (6.1%)	2668 (6.1%)	14 (4.1%)	6 (8.6%)	3 (6.1%)
37-40	37,229 (84.3%)	36,841 (84.3%)	290 (85.6%)	57 (81.4%)	41 (83.7%)
> 40	4227 (9.6%)	4180 (9.6%)	35 (10.3%)	7 (10.0%)	5 (10.2)
Gestational age (weeks)	39 (38-40)	39 (38-40)	39 (39-40)	39 (38-39)	39 (39-40)
Birth Weight (g)	3420 (3130-3720)	3420 (3130-3720)	3480 (3170-3800)	3245 (3000-3515)	3440 (3160-3790)
Age at screening (hours)	20 (15.0-24.0)	20 (15.0-24.0)	20 (16.0-24.0)	19.5 (15.8-24.0)	19 (15.5-24.0)
Delivery method					
Natural birth	24,159 (54.7%)	23,894 (54.7%)	205 (60.4%)	35 (50.0%)	25 (51.0%)
Cesarean section	19,946 (45.2%)	19,754 (45.2%)	133 (39.2%)	35 (50.0%)	24 (49.0%)
Natural birth to cesarean section	42 (0.1%)	41 (0.1%)	1 (0.2%)	0	0

There were 498 newborns identified with CHD initially – 27 by POX screening and 471 by cardiac auscultation – with an overall screening rate of 1.13% among all 44,147 livebirths. Of these cases, 458 neonates were confirmed by echocardiography, making the overall diagnostic rate of CHD via echocardiogram 92.0% (including 253 male babies and 245 female babies). The screen results were presented in Table 2. The most common types of CHD were PDA (34.3%), ASD (20.5%), VSD (8.3%), and combined complications (34.5%). Rare CHD included COA, CTA PAPVC, TAPVC, TGA, and TOF, accounting for a total of 2.2% cases. Mild CHD accounted for 74% of all cases, followed by moderate CHD (15.3%), and severe CHD (10.7%). We also conducted chart review of newly diagnosed CHD in 2022, and didn’t identify additional CHD case after cross-checking with the results of newborn screening.

**Table 2:** Result of CHD cases

CHD Type	Mild CHD	Moderate CHD	Severe CHD	
	Case counts (%)	Case counts (%)	Case counts (%)	Total case counts (%)
ASD	70 (15.3)	24 (5.2)	0 (0)	94 (20.5)
ASD&PDA	76 (16.6)	13 (2.8)	10 (2.2)	99 (21.6)
COA	0	1 (0.2)	0	1 (0.2)
CTA	0	1 (0.2)	0	1 (0.2)
PAPVC	0	2 (0.4)	0	2 (0.4)
PDA	127 (27.7)	9 (2.0)	21 (4.6)	157 (34.3)
TAPVC	0	0	3 (0.7)	3 (0.6)
TGA	0	0	2 (0.4)	2 (0.4)
TOF	0	0	2 (0.4)	2 (0.4)
VSD	27 (5.9)	7 (1.5)	4 (0.9)	38 (8.3)
VSD&ASD	14 (3.1)	5 (1.1)	2 (0.4)	21 (4.6)
VSD&ASD&PDA	9 (2.0)	3 (0.7)	2 (0.4)	14 (3.1)
VSD&PDA	16 (3.5)	5 (1.1)	3 (0.7)	24 (5.2)
Total	339 (74.0)	70 (15.3)	49 (10.7)	458 (100)

Of the 458 confirmed CHD subjects, cardiac auscultation alone detected majority of 438 CHD cases (95.6%) and POX only screened 20 (4.4%) cases. However, no single incidence of CHD was recognized by both auscultation and POX screening (Table 3). A potential explanation for this result is that auscultation detected most of the common types of CHD neonates, but POX detected the rare, yet critical CHD types, including COA, CTA, PAPVC, TAPVC, TGA, and TOF. A similar finding was observed in a new set of data from 2020 (data not shown).

**Table 3:** Detection for CHD types

		Detection rate		
CHD types	N	Auscultation alone	Pulse oximetry alone	Auscultation or pulse oximetry
**Study Period Data**				
ASD	94	94	0	0
ASD&PDA	99	99	0	0
COA	1	0	1	0
CTA	1	0	1	0
PAPVC	2	0	2	0
PDA	157	154	3	0
TAPVC	3	0	3	0
TGA	2	0	2	0
TOF	2	0	2	0
VSD	38	35	3	0
VSD&ASD	21	20	1	0
VSD&ASD&PDA	14	12	2	0
VSD&PDA	24	24	0	0
In Total	458	438	20	0
**First Half of 2020 Data**				
ASD	6	6	0	0
VSD	8	8	0	0
PDA	10	10	0	0
ASD&VSD	2	2	0	0
VSD&PDA	2	2	0	0
TOF	1	0	1	0
TAPVC	1	0	1	0
In total	30	28	2	0

POX screening alone had a low accuracy of 74.07% in positive predict value (PPV). Auscultation alone functioned well in terms of PPV and negative predict value (NPV) (92.99 and 99.95%, respectively), but the addition of POX improved the overall screening performance to 100% NPV (Table 4).

**Table 4:** Screening accuracy for CHD newborns (*n* = 44,147)

	Pulse oximetry alone	Auscultation alone	Auscultation or pulse oximetry
True positives	20	438	458
False negatives	438	20	0
False positives	7	33	40
True negatives	43,682	43,656	43,649
Sensitivity	4.37%	95.63%	100.00%
Specificity	99.98%	99.92%	99.91%
Positive Predict Value	74.07%	92.99%	91.97%
Negative Predict Value	99.01%	99.95%	100%

As PDA and ASD were not included in prenatal screening by ultrasonography, they were excluded when tracing back prenatal ultrasound screening. Of the remaining 108 cases, 37 (34.3%) fetuses were diagnosed with CHD, including 35 cases of VSD, 1 case of COA, and 1 case of TOF (Table 5).

**Table 5:** Prenatal trace back of confirmed CHD cases excluding PDA and ASD cases (*n* = 108)

Conditions	Case counts	Percentage (%)
Prenatal identification of CHD		
VSD	35	32.41
COA	1	0.93
TOF	1	0.93
Prenatal identification of non-CHD	51	47.22
Unclear ultrasound image	3	2.78
No prenatal screening	14	12.96
Lost to trace back	3	2.78

## Discussion

This was the first retrospective report of large-scale clinical implementation of combined POX and cardiac auscultation in routine CHD screening for all newborn infants within 24 hours after delivery. Within a two-year period, we identified 458 CHD neonates from 44,147 live births, of which 74% neonates are diagnosed with mild CHD, 15.3% with moderate CHD, and 10.7% with major CHD. POX in conjunction with clinical auscultation resulted in a high detection rate of serious CHD.

The most intriguing finding in this clinical evaluation was that there was no overlap of CHD spectrums between POX detection and auscultation detection. In contrast to common CHDs detected by auscultation, POX-detected CHDs were rare and critical, including COA, CTA, PAPVC, TAPVC, TGA, and TOF, which were consistent with the primary and second target lesions presenting at least mild hypoxemia during the neonatal period [11]. This observation was different from two previous research with significant overlaps between two screening techniques [8, 12]. The dynamic changes of pulmonary arterial pressure in newborns in combination of all screenings conducted within the first 24 hours after delivery likely contributed to the difference between our findings and the previous observations. In fetal period, pulmonary circulation is not in use. Instead, it is occupied by amniotic fluid and full of pressure. After birth, because postnatal circulation requires inhalation of air, lung aeration initiates and clears the airway fluid, accompanied by decreasing pulmonary arterial pressure from 60 mmHg at birth to about 30 mmHg at 24 hours of age [13–15]. Since all of our screenings were conducted within the first 24 hours of delivery, which was different from the 72-hour window used in previous studies, it might be difficult to detect abnormal heart murmur when pulmonary arterial pressure still maintained relatively high levels. Moreover, in one of two previous studies with a large population of 167,190 asymptomatic newborn infants, most POX-recognizable CHD cases were only detected via auscultation [12]. Since this study was a multicenter research investigating the accuracy and feasibility of implementing cardiac auscultation and POX as a screening method in China, there might be large variations in the POX measurement among different hospitals studied for different researches, resulting in low detection rate by POX in other studies. Our findings manifested the complementary aspects of POX and auscultation in early screening of CHD among newborns, shedding light on the effectiveness of combined use of both methods in comprehensive neonatal screening, especially regions lacking echocardiologists.

Meanwhile, the commonality of certain types of CHD was different: PDA, VSD, and ASD accounting for 97.6% of total CHD cases in our study and ~ 91.1% in previous studies. The difference in incidence rate and the CHD spectrum could be explained by differences in prenatal management among different hospitals [8, 12]. Tertiary referral hospitals have more medical resources and better prenatal screening and management systems. Pregnant women registered at these hospitals often received additional prenatal ultrasound screening, which can increase the chance of prenatal diagnosis of major CHD, usually followed with termination of pregnancy. Contrasting to previous multicenter researches which collected data from a wide range of hospitals, our study was conducted at a single, tertiary referral hospital which had better prenatal management systems, prenatal screenings and adequately trained medical staffs [16].

Some mild congenital defects, including small muscular VSD, ASD and PDA, would become less noticeable or even close spontaneously without intervention [17]. Owing to the natural closure, these neonates should not be included when calculating CHD incidence, or if counted, it will increase the incidence rate at different stages. Moreover, due to limited resource, our study only considered observable CHD cases before discharge, whereas other studies offered follow up statistics among neonates after discharge. Thus, previously misdiagnosed or undiagnosed neonates would be determined to have CHD and added to the total CHD incidence later in the study. Furthermore, some studies would exclude CHD cases that were screened out in prenatal ultrasonography and confirmed in postnatal echocardiography, and symptomatic CHD neonates such as cyanotic subjects would not be included, either. As a whole, these limitations explain the observable differences in overall CHD occurrence and the major type of diagnosed CHD between our study and the previous studies.

Since this was a retrospective report of large-scale clinical implementation of combined POX and cardiac auscultation in routine CHD screening and we didn’t conduct follow-up investigation, it was possible some CHD infants were missed in screening. We therefore conducted a chart review of newly diagnosed CHD in 2022, and didn’t identify additional CHD case after cross-checking with the results of newborn screening. We presumed that the studied hospital was the major tertiary hospital for pediatric referrals, and CHD children missed in newborn screening could be identified from chart review. Although it was possible that missed CHD children could be referred to another hospital, the number of missed CHD cases should be small.

## Conclusions

Our study demonstrates that within the first 24 hours after birth, when auscultation and POX combined in CHD screening, it can generate quick and accurate outcomes in an economic way, which can benefit facilities in low-income area and hospitals short of ward resource.

## Data Availability

The datasets used and/or analyzed during the current study available from the corresponding author on reasonable request.

## Abbreviations

ASD: Atrial septal defect
CCHD: Critical congenital heart disease
CHD: Congenital heart disease
COA: Coarctation of the aorta
CTA: Cor triatriatum
NPV: Negative predict value
PAPVC: Partial anomalous pulmonary venous connection
PDA: Patent ductus arteriosus
POX: Pulse oximeter
PPV: Positive predict value
SpO2: Oxygen saturation
TAPVC: Total anomalous pulmonary venous connection
TGA: Transposition of the great arteries
TOF: Tetralogy of Fallot
VSD: Ventricular septal defect
